# Zebrafish Models of Autosomal Recessive Ataxias

**DOI:** 10.3390/cells10040836

**Published:** 2021-04-08

**Authors:** Ana Quelle-Regaldie, Daniel Sobrido-Cameán, Antón Barreiro-Iglesias, María Jesús Sobrido, Laura Sánchez

**Affiliations:** 1 Department of Zoology, Genetics and Physical Anthropology, Faculty of Veterinary Science, Universidade de Santiago de Compostela, 27002 Lugo, Spain; ana.quelle@usc.es; 2 Department of Functional Biology, CIBUS, Faculty of Biology, Universidade de Santiago de Compostela, 15782 Santiago de Compostela, Spain; ds918@cam.ac.uk (D.S.-C.); anton.barreiro@usc.es (A.B.-I.); 3 Instituto de Investigación Biomédica de A Coruña (INIBIC), Servicio Galego de Saúde, 15006 A Coruña, Spain; ssobrido@gmail.com; 4 Preclinical Animal Models Group, Health Research Institute of Santiago de Compostela (IDIS), 15706 Santiago de Compostela, Spain

**Keywords:** zebrafish, hereditary recessive ataxias, neurodegenerative disorders, genetic edition

## Abstract

Autosomal recessive ataxias are much less well studied than autosomal dominant ataxias and there are no clearly defined systems to classify them. Autosomal recessive ataxias, which are characterized by neuronal and multisystemic features, have significant overlapping symptoms with other complex multisystemic recessive disorders. The generation of animal models of neurodegenerative disorders increases our knowledge of their cellular and molecular mechanisms and helps in the search for new therapies. Among animal models, the zebrafish, which shares 70% of its genome with humans, offer the advantages of being small in size and demonstrating rapid development, making them optimal for high throughput drug and genetic screening. Furthermore, embryo and larval transparency allows to visualize cellular processes and central nervous system development in vivo. In this review, we discuss the contributions of zebrafish models to the study of autosomal recessive ataxias characteristic phenotypes, behavior, and gene function, in addition to commenting on possible treatments found in these models. Most of the zebrafish models generated to date recapitulate the main features of recessive ataxias.

## 1. Introduction

Hereditary ataxias are a heterogeneous group of rare neurodegenerative disorders caused by progressive degeneration of the cerebellum and spinocerebellar tracts resulting in loss of balance and coordination. Classification of hereditary ataxias has been problematic due to phenotype overlap and the broad number of syndromes that manifest ataxia as a common symptom, such as hereditary spastic paraplegias and polyneuropathies. However, the development of novel molecular techniques has made it possible to classify ataxias based on genetics [1]. Ataxias can be divided into autosomal dominant, autosomal recessive and X-linked ataxias [2,3].

Unlike autosomal dominant ataxias [4], autosomal recessive ataxias have been less well studied. Autosomal recessive ataxias are more difficult to classify because, contrary to autosomal dominant ataxias, they are not organized with a numerical naming system. The term spinocerebellar ataxia autosomal recessive (SCAR) was recently used to designate novel ataxias, but it is based only on locus discovery and it does not include the previously identified and more frequent recessive ataxias. In addition, many recessive multisystemic or complex metabolic disorders present ataxia as a symptom [2,3]. There are more than 100 loci related with recessive ataxias. The development of next generation sequencing technology helped to classify and discover new recessive ataxias [5]. Autosomal recessive ataxias are a group of complex neuronal disorders usually showing early onset, involving central and peripheral nervous systems. They often affect other systems and organs and cause—among others—symptoms such as: peripheral sensorimotor neuropathy; pyramidal and extrapyramidal involvement; oculomotor defects; retinopathy; cognitive impairment; metabolism abnormalities; seizures and hypogonadism [2,6]. The pathogenesis of autosomal recessive ataxias is associated with loss of function of the related proteins, which are involved in a broad number of pathways such as: mitochondrial metabolism and function; metabolic homeostasis; DNA repair; the cell cycle; RNA processing; protein folding; synaptic morphology and the synaptogenesis of Purkinje cells [3,7,8].

The use of animal models of neurodegenerative disorders can, at least partially, recapitulate the human disease to help unravel the molecular and cellular mechanisms responsible and represent a first step in developing therapies for diseases that do not yet have available treatments [9]. For example, in ataxias, Purkinje cell degeneration and motor deficits were demonstrated in several mice models [10].

The zebrafish (*Danio rerio*) shares 70% of its genome with humans [11] and is an emerging animal model that is gaining popularity in the study of a wide range of medical conditions, such as cancer [12], neurogenetic diseases [13], psychiatry disorders [14], cardiovascular illnesses [15] and traumatic injuries [16,17]. Its many advantages include: easy genetic manipulation; the capacity to perform high throughput drug and genetic screening; optical transparency that allows for visualizing the central nervous system (CNS) and cellular processes. In addition, behavioral patterns are measurable and easy to evaluate, including high order behaviors such as memory, conditioned responses and social behaviors [18]. The organization of the CNS of the zebrafish is similar to mammals, including humans. Moreover, most genes related to neural activity or development show similar functions in mammals and zebrafish. On the other hand, the zebrafish model has the advantage of having a reduced CNS complexity, which simplifies functional studies [19].

**Figure 1:**
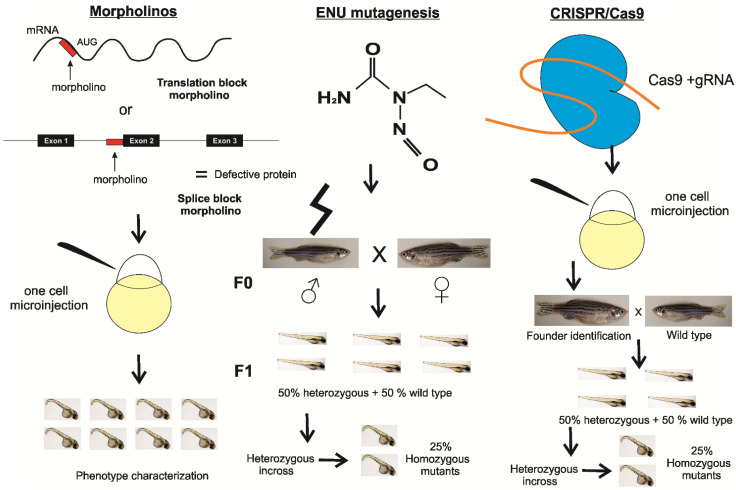
Main techniques for generating zebrafish loss of function models.

The development of zebrafish loss of function models is easily achieved by transient knock-down with antisense morpholinos that block translation or splicing [20]. Permanent knock-out models are created with mutagens such as N-ethyl-N-nitrosourea (ENU) [21], transgenesis or genetic edition via the use of TALENs (Transcription activator-like effector nucleases), zinc finger nucleases or the recently discovered CRISPR/Cas9 techniques [22] (Figure 1).

In this review, we used the Bird classification [23] of ataxias (which includes recently discovered ataxias) to search zfin (https://zfin.org/ (accessed on 27 January 2021)) and PubMed (https://pubmed.ncbi.nlm.nih.gov/ (accessed on 27 January 2021)) databases for zebrafish models targeting genes that cause recessive disorders with cerebellar ataxia among their clinical manifestations. These data are compiled in Supplemental Table S1. We discuss the zebrafish models created for the more common recessive ataxias, recessive ataxias that are relatively more uncommon (only present in a few families), and recessive disorders that cause abnormalities of cerebellar development and prominent ataxia. The molecular basis and relevant clinical data of the diseases discussed in the present review are summarized in Table 1 (more frequent autosomal recessive ataxias in green, less frequent autosomal recessive ataxias in blue and recessive inherited disorders related to ataxia in orange).

## 2. Materials and Methods

Articles were found in the zfin database (https://zfin.org/ (accessed on 27 January 2021)) by searching for zebrafish models of genes that cause recessive ataxias as described in the classification of Bird [23] and some recessive disorders that have prominent ataxia.

The PubMed database (https://pubmed.ncbi.nlm.nih.gov/ (accessed on 27 January 2021)) was used to find additional models related to these diseases by searching for “zebrafish” and “name of the disease”. The articles included in this review were published before 27 January 2021.

## 3. Results

**Figure 2:**
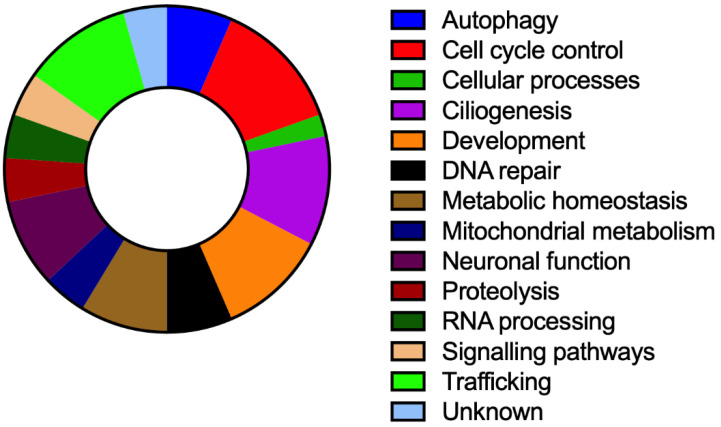
Main functions of genes related to recessive ataxias.

The genes responsible for the diseases discussed here are implicated in a broad number of signaling pathways and cellular processes that are summarized in Figure 2. In the following sections, we will review the main findings obtained with zebrafish models for each of these genes.

### 3.1. Autosomal Recessive Cerebellar Ataxias

- Ataxia-telangiectasia (AT).

AT is an infantile onset multisystemic disease produced by mutations in the *ATM* gene (ataxia-telangiectasia mutated), which is involved in cellular responses to DNA damage and cell cycle control [24].

The first ataxia-telangiectasia zebrafish model was generated with morpholinos and reproduced the role of *ATM* in the DNA damage response [55]. *Atm* morphants exhibit normal development but when a radiation dose of 8 Gy was applied to 6 hpf *atm* morphants, abnormal development with retardation was observed. *Atm* irradiated morphants exhibited no pigmentation, a lack of yolk extension, and extreme ventral body curvature and died by 72 hpf. Higher amounts of *atm* morpholino, combined with radiation, increased mortality of embryos by 48 hpf and induced more severe curvature and absence of yolk extension phenotypes. This phenotype caused by irradiation was not seen in embryos injected with a control morpholino, which means that deficiency in *atm* leads to increased radiosensitivity [55].

Recently, an *atm* zebrafish mutant was developed which did not show neurodegenerative defects but developed exclusively as infertile males [56]. Infertility is a characteristic of some AT patients. Disabling the apoptosis pathway with a *p53* mutation rescued the all-male phenotype, but not infertility. In addition, *atm*-*p53* double mutants showed quick tumor formation, which suggests that *atm* acts as a tumor suppressor and is involved in maintaining genomic stability. Surprisingly, *atm* mutant embryos did not display the radiosensitivity observed in *atm* morphant embryos. This is probably due to compensation mechanisms [56]. These results indicate that zebrafish models of *atm* can be used to analyze the function of ATM in the DNA damage response but not for neurodegeneration.

- Boucher–Neuhäuser syndrome and spastic paraplegia type 39 (SPG39).

Mutations in the *PNPLA6* (patatin like phospholipase domain containing 6) gene cause SPG39 and Boucher–Neuhäuser syndrome which express similar symptoms [25]. Other syndromes that have been related with mutations in *PNPLA6* include: (i) Oliver–McFarlane syndrome, the features of which consist of trichomegaly, congenital hypopituitarism and retinal degeneration with choroidal atrophy; (ii) Laurence–Moon syndrome, which presents in a similar manner, though with progressive spinocerebellar ataxia and spastic paraplegia, and without trichomegaly [57].

*Pnpla6* zebrafish morphants have been shown to exhibit a curly tail that is dose-dependent. The head–tail distance was shorter in morphants and they also had abnormal eye development and reduced otic vesicle size. Morphant eyes were smaller than in controls and some of them had unclosed eye fissures [57,58]. In addition, some *pnpla6* morphants showed midbrain–hindbrain boundary defects, swelling of the pericardium, reduced heart rate and reduced blood flow [58]. Normal *PNPLA6* human mRNA was able to rescue the phenotype but not *PNPLA6* human mRNA without the catalytic domain. This means that the phenotype in *pnpla6* morphants was due to reduced esterase activity [58]. Likewise, co-injection with human mRNA with known mutations of SPG39, Oliver–McFarlane syndrome or Laurence–Moon syndrome, did not rescue the phenotype [57]. Motor neurons and spinal motor neuron numbers were reduced in *pnpla6* morphant zebrafish. Aberrant motor axon branching was also observed, which seemed to be truncated and failed to join the ventral muscles. An increase in apoptosis was observed in this model, especially in the CNS, that was not rescued with co-injection of *p53* morpholino. Cell cultures of *pnpla6* morphant spinal neurons were performed to confirm that the cause of impaired motor neuron development was not the presence of a deformed body. Chimeric embryos transplanted with one cell stage *pnpla6* morphant cells also developed axon defects. Mauthner neurons, interneurons and sensory neurons showed no morphological differences in *pnpla6* morphants. BMP (bone morphogenetic protein) signaling was studied by blocking the kinase activity of BMP receptors with dorsomorphin, which resulted in phenotype rescue in *pnpla6* morphants. This indicates that BMP was overexpressed in *pnpla6* knock-down and that this caused at least part of the morphant phenotype [58]. Although these results revealed the neurological consequences of *pnpla6* dysfunction and a relation with BMP overexpression, it is necessary to develop a mutant zebrafish model to see if this will reproduce the phenotype observed with morpholinos.

- Marinesco–Sjögren syndrome.

Marinesco–Sjögren syndrome is a multisystemic early onset disorder caused by mutations in the *SIL1* (SIL1 Nucleotide Exchange Factor) gene, which encodes a nuclear exchange factor for the endoplasmic reticulum resident chaperone BiP (binding immunoglobulin protein) [26].

Zebrafish morphants for *sil1* showed abnormal shape and small eyes. Birefringence assays revealed abnormal skeletal muscle structure at 4 dpf in morphant embryos. The phenotype was rescued by the co-injection of *sil1* wild type mRNA. Histological examination revealed deformed beta-dystroglycan expression at myosepta with a less clear V-shaped structure and disturbed formation in *sil1* morphants. Reduced numbers of Purkinje cells were observed in *sil1* morphant embryos in comparison with controls. Interestingly, *sil1* morphants displayed an increase in levels of BiP, the lipidated form of Lc3 (light chain 3) and caspase 3, which are markers associated with endoplasmic reticulum stress, autophagy and apoptosis, respectively [59].

*Sil1* deficiency in peripheral nerves and neuromuscular junctions was also studied using *sil1* morpholino knock-down in zebrafish. *Sil1* morphants demonstrated disrupted synapse formation along the vertical myosepta, with remarkable disorganization of presynaptic branching across the muscle fibers of the myotomes. Analysis of myelinating Schwann cells did not show significant differences between *sil1* morphants and control zebrafish [60].

These morphant models revealed neural damage, abnormalities in the skeletal muscle and neuromuscular junctions, and activation of endoplasmic reticulum stress, autophagy and apoptosis, which are features of Marinesco–Sjögren patients. The development of a mutant zebrafish model will assist in the search for treatments and confirm the morphant phenotypes.

- Spinocerebellar ataxia autosomal recessive type 20 (SCAR20).

SCAR20 is caused by mutations in the sorting nexin 14 gene (*SNX14*), which is required for autophagosome clearance [27].

*Snx14* zebrafish morphants displayed loss of neural tissue volume and reduced number of Purkinje cells that was rescued with co-injection with human *SNX14* mRNA. In addition, morpholino injection in a reporter line that expressed GFP in the hindbrain revealed a significant reduction in hindbrain size in morphant embryos. An increase in apoptosis across the neural tissue was also seen. Transmission electron microscopy analysis of *snx14* morphant neural cells also revealed an accumulation of autophagic structures. These data suggest that *snx14* mutations caused neuronal cell death associated with impaired autophagic degradation and apoptosis [27].

Surprisingly, a more recent study using *snx14* morpholinos in zebrafish did not detect morphological defects or motor neuron abnormalities. In the same study, an *snx14* zebrafish knock-out model was examined, and no morphological or neuronal abnormalities were detected, not even when maternal zygotic mutants were analyzed. Behavioral analysis also showed that mutant *snx14* zebrafish did not develop a locomotor phenotype. As *SNX14* mutations had been reported to disrupt neutral lipid metabolism, lipids from 4 dpf zebrafish were extracted and analyzed through total fatty acids lipidomic profiling. It was observed that total fatty acids from neutral lipids (both saturated and unsaturated) were elevated in *snx14* knock-out embryos in comparison with wild type and heterozygous embryos. These results confirm that SNX14 has a conserved role in lipid biogenesis [61].

The discrepancies in these studies could be because Bryant et al. [61] used sub toxic doses of morpholinos. Morpholinos are only considered reliable when a mutant model supports morpholino findings due to the previously observed off target effects of morpholinos [62]. In addition, the premature stop codon of mutants resulted in a nonsense mediated decay that upregulated homologous gene sequences that rescue the phenotype. The consequence of the mutation at the protein level could not be demonstrated in zebrafish because there was no SNX14 antibody available that could react with the corresponding zebrafish protein [61].

- Ataxia with isolated vitamin E deficiency (AVED).

AVED is caused by mutations in the α tocopherol transfer protein (*TTPA*) gene which is responsible for the distribution of vitamin E [28].

*Ttpa* is expressed in the zebrafish brain, eye and tailbud. Experiments with morpholinos in zebrafish showed significant developmental defects at 1 dpf along the anterior/posterior axis, with truncated tail and brain and eye malformations. However, time lapse analysis reported normal development during blastula formation, epiboly and gastrulation until 12 hpf, when the *tppa* morphant eye began to display tissue darkening, indicating the initiation of improper head growth. Co-injection with a *p53* morpholino did not affect the phenotype [63].

Previous studies with a vitamin E-deficient diet in adult zebrafish caused a decreased startle response that suggested the occurrence of neurological defects. Embryos produced by zebrafish E depleted adults displayed increased mortality and morphological abnormalities such as cranial malformations, a bent anterior–posterior axis, pericardial edema, swim bladder malformations and yolk-sac edema [64,65]. Surviving vitamin E deficient embryos of 24 hpf had somite malformations and stunted fin formation. At 48 hpf embryos developed acute pericardial and yolk sac edema. Collagen sheath notochord markers col2a1a (collagen, type II, alpha 1a) and col9a2 (collagen, type IX, alpha 2) showed bent axes. Surprisingly, it was demonstrated that a vitamin E deficient diet did not affect the localization or expression of *ttpa* but affected brain structure. In addition, *tppa* was found to be expressed at 24 hpf in the midbrain–hindbrain boundary and pax2a (paired box 2a) (which defines the midbrain–hindbrain boundary) expressed a diffused signal at 12 and 24 hpf [65]. Future studies with mutants may support the discoveries made with morpholinos and with a vitamin E deficient diet.

- Wolfram syndrome (WFS).

WFS is a juvenile progressive neurodegenerative disorder caused by mutations in *WFS1*, which encodes the wolframin protein. This is an endoplasmic reticulum membrane-embedded protein related to membrane trafficking, endoplasmic reticulum stress and calcium homeostasis [29,30].

Zebrafish have two paralogous genes of *WFS1*, *wfs1a* and *wfs1b*. The first knock-down of *wfs1b* was created during an assessment of genes that are relevant to diabetes type 2. *Wfs1b* knock-down caused a reduction in pancreatic β-islet cells and reduced insulin expression, suggesting that *WFS1* is involved in glucose homeostasis [66].

A broader study of zebrafish models was performed by Cairns [67]. Morpholinos for *wfs1a* and *wfs1b* caused abnormal eye, brain and otoliths, although the *wfs1b* phenotype was more pathological. Co-injection of the two *wfs1* morpholinos caused lethality in most embryos and *p53* morpholino co-injection did not rescue the phenotype. CRISPR/Cas9 models of *wfs1a* and *wfs1b* genes displayed delayed development and smaller otoliths. *Wfs1* knock-outs, particularly the *wfs1b* knock-out, showed significant delay in neuronal growth. At 24 hpf, *wfs1b* displayed shortened motor axons that seemed to be corrected at 48 hpf. Expression of acetylcholine receptors developed normally in both *wfs1* knock-outs, indicating that muscular growth was normal. Acetylcholinesterase activity was reduced in *wfs1a* and *wfs1b* knock-out embryos, which suggested reduced brain activity, but only was reduced in the *wfs1b* knock-out at 12 months, suggesting defective neurogenesis in *wfs1b* knock-out fish. Moreover, mutants had a smaller cerebellum than the wild types. Spontaneous movements of *wfs1b* knock-out zebrafish were significantly higher at 24 hpf and significantly lower at 48 hpf, as compared to both controls and *wfs1a* knock-outs. As no differences were detected in the muscle fiber structure, this was associated with problems related to neuronal signaling in response to tactile stimulation. Mitochondrial trafficking was affected in both *wfs1* knock-outs and mitochondrial respiration was reduced in the *wfs1b* knock-out. Mitochondrial complex I protein expression was reduced in *wfs1a* and *wfs1b* knock-out whole embryos and muscle and also in the brain in *wfs1b* knock-out embryos. However, Ca^2+^ uptake and mitochondrial DNA levels remained normal in both *wfs1* knock-outs. Heat shock—to induce endoplasmic reticulum stress—increased the death rate in the two *wfs1* knock-outs, suggesting increased apoptosis due to increased endoplasmic reticulum stress response. BiP protein expression was shown to be upregulated in both heat shocked and untreated *wfs1* knock-outs. *Wfs1b* knock-out adult zebrafish displayed less locomotion when they were isolated in order to record their behavior, which suggested increased anxiety. *Wfs1b* knock-out adults had a significantly lower blood glucose level and lower fertility than *wfs1a* and wild type fish. In addition, both knock-outs had a significantly lower number of retinal ganglion cells than wild type fish at 4 months and this number decreased even more at 12 months. *Wfs1b* retinal ganglion cell numbers were also significantly lower than in *wfs1a*. Less eye movement and a thinner optic nerve were also observed in the *wfs1b* knock-outs at 12 months. The fact that *wfs1b* displayed the most severe phenotype, replicating some of the symptoms of wolfram syndrome patients, could be due to its expression in eyes and neural tissue, unlike *wfs1a* which was only found to be expressed in muscle tissue [67]. The use of these models reproducing the pathological mechanisms of patients with WFS may help to find a therapy to Wolfram syndrome.

### 3.2. Less Frequent Autosomal Recessive Ataxias

- Polyneuropathy, hearing loss, ataxia, retinitis pigmentosa and cataract (PHARC).

PHARC is produced by mutations in *ABHD12*, which encodes the abhydrolase domain containing protein 12, an enzyme related to endocannabinoid and phospholipid metabolism and that has essential functions in the central and peripheral nervous systems [31].

A morpholino for the *ABHD12* gene was generated as part of a study on neuropathy candidate genes related to Charcot–Marie–Tooth disease. *Abhd12* morphant embryos had aberrant motor neuron axon extension, pathfinding and branching, with an abnormal morphology of peripheral neurons [68].

In a more recent study, *Abhd12* zebrafish morphants injected with a splice block morpholino displayed two phenotypes: mild and moderate. The moderate phenotype included severe microphthalmia, microcephaly and reduced body length due to a significant reduction in head size. From 3 dpf, both morphant phenotypes did not have spontaneous free-swimming activity. The moderate phenotype did not display any locomotor activity in a touch-evoked escape response and mild phenotype animals exhibited impaired locomotor activity similar to ataxia. None of these phenotypes were rescued after *p53* morpholino co-injection. Axonogenesis and motor neuron axon outgrowth was normal in *abhd12* morphants at 3 dpf. However, myelination of the axonal tracks was severely impaired in *abhd12* morphants at 5 dpf. Neuromasts suffer a large reduction in the anterior and posterior lateral line and auditory system in comparison with controls. Normal Purkinje cell differentiation was observed, while the morphology of the Purkinje cell layer was affected. A strong impairment of retinal architecture and stratification, absence of lens clarification and a high decrease in retinotectal projections was also found in *abhd12* morphants. Injection of a translation block morpholino caused a severe phenotype with exacerbation of morphological symptoms, a curved tail and the absence of spontaneous swimming. Co-injection with *abhd12* mRNA of wild type zebrafish embryos or human *ABHD12* mRNA rescued the phenotype of morphant embryos. However, co-injection with human *ABHD12* mRNA encoding one missense or nonsense mutation associated with PHARC could not restore the normal phenotype [69]. The molecular consequences of blocking translation seemed to be worse than when blocking mRNA splicing. This may be due to the different biological role of some parts of the truncated protein.

These models reproduced several of the symptoms of PHARC patients, such as ataxia, disruption of retinal architecture and a reduction in mechanosensory hair cells in the inner ear and lateral line. However, it is necessary to generate mutant models to confirm that these findings were not caused by off-target effects and to search for therapies.

- Cayman ataxia.

Cayman ataxia is a recessive congenital ataxia restricted to the Grand Cayman Island. Cayman ataxia is caused by mutations in the *ATCAY* gene, which encodes a neuronal protein called Caytaxin [32].

*Atcay* morphants displayed reduced motor axon length in a dose-dependent manner at 72 hpf. This phenotype was rescued with mouse *atcay* mRNA and also by mouse *VAchT* (vesicular acetylcholine transporter) mRNA, which acts downstream of acetylcholine synthesis and release. This indicates that *atcay* regulates neurite outgrowth through cholinergic signaling. Interestingly, knock-down of *chat* (choline acetyltransferase, which produces acetylcholine) or *acl* (ATP citrate lyase, an enzyme that produces acetyl-CoA for lipogenesis, cholesterol genesis and acetylcholine synthesis in the citosol) genes also caused shorter motor neuron axons. Primary motor neurons from the 18 somite stage of zebrafish embryos were isolated. Neurite outgrowth could be rescued by supplying carbachol, a cholinergic agonist. However, atropine (a muscarinic receptor) application in *atcay*, *acl* and *chat* knock-downs inhibited neurite outgrowth in vitro, this phenotype was not rescued by carbachol. These data confirm that *atcay* functions through muscarinic acetylcholine receptors and the MAPK (mitogen-activated protein kinase) pathway to regulate neurite outgrowth. The subcellular location of Chat changed in *atcay* and *acl* morphants, with Chat accumulating in the cell bodies of motor neurons. Chat accumulation in *atcay* morphants could be rescued with mice *Atcay* mRNA co-injection. Knock-down of *chat* in zebrafish inhibited acetylcholine neurotransmission and caused enlargement of acetylcholine receptor clusters to compensate for the loss of cholinergic signaling. Interestingly, *atcay* and *acl* morphants also showed an enlargement of acetylcholine receptor clusters. Enlargement of the acetylcholine receptor clusters in *atcay* morphants was rescued by *VAchT* mRNA co-injection. In addition, loss of acetylcholine signaling also affected the development of muscle fibers in *atcay* morphants, which showed abnormal structures of slow and fast muscles. These data suggest that Atcay regulates cholinergic signaling at the neuromuscular junction and that cholinergic signaling is required for the proper development of neuromuscular connections. Motor behavior was also affected in *atcay* morphants, which displayed a deteriorated escape response after touch stimulus and a delay in spontaneous movement. This defect was rescued by *Atcay* and *Vacht* mRNA co-injection. *Acl* and *chat* morphants had similar behavior phenotypes [70]. This study helped to elucidate that *atcay* has a role in motor neuron development and this model was used to show that carbachol treatment rescues neurite outgrowth in *atcay* morphant cells. Thus, this study might be a first step in the development of a treatment that requires confirmation through the use of an *atcay* mutant.

- Spinocerebellar ataxia autosomal recessive type 20 (SCAR25).

Mutations in the *ATG5* gene are related with congenital childhood ataxia with mental retardation, and developmental delay. ATG5 (autophagy protein 5) is an E3 ubiquitin ligase that is essential for autophagosome elongation. Patients with mutations in *ATG5* show defects in autophagy [33].

*Atg5* morpholino knock-down in zebrafish resulted in high mortality (between 40 and 60%). Zebrafish showed: smaller and incomplete head and eyes; otolith deformities; an abnormal heart structure with less blood and congestion in the ventral common cardinal vein; smaller body; decreased tail and yolk extension; somite deficiency. Therefore, *atg5* seems to be involved in the formation of essential embryonic structures, mainly in the neural system. The phenotype was rescued with co-injection of wild type *atg5* mRNA. Interestingly, *atg5* overexpression led to 30% mortality, development retardation, an absence of body pigments, small otoliths, ventralized optic vesicles, reduced brain development, insufficient differentiation of the trunk and abdomen, and diminished clarity of the yolk and yolk extension. Surprisingly, *atg5* mRNA levels were upregulated in morphants and downregulated after *atg5* overexpression. In contrast, the *atg5* protein was reduced in the morphants and increased in embryos with mRNA overexpression. These data suggest that the Atg5 protein can regulate *atg5* transcription in a feedback inhibitory loop. In addition, LC3B (light chain 3B), an autophagy marker, showed reduced protein expression in *atg5* morphants and increased expression upon *atg5* mRNA overexpression, which demonstrated that the autophagy pathway is regulated by *atg5* during neural development in zebrafish embryos. Analysis of mRNA expression of several genes involved in neuronal development showed upregulation in *atg5* morphant embryos and downregulation of the same genes in embryos injected with *atg5* mRNA. Rapamycin treatment, which is a reagent for autophagy induction, produced a similar embryonic phenotype to that of *atg5* morphants [71].

A more recent study using morpholinos of the autophagy related genes *atg5*, *atg7* and *becn1* in a zebrafish autophagy reporter line showed inhibition of autophagosome accumulation in vivo. Immunoblots of protein lysates from primary cell cultures of the three morphants revealed blocked Lc3-II conversions, which means that autophagy was inhibited. *Atg5*, *atg7* (autophagy protein 7) and *becn1* (beclin1) morphants displayed a similar phenotype at 2 dpf, with small heads and eyes, twisted body shapes and cardiac defects such as pericardial edema, defective blood flow through the heart, defective heart looping, enlarged atria and a linearized heart. Knock-down of these genes in a *p53* zebrafish mutant line resulted in the same phenotype. *Atg5*, *atg7* and *becn1* 1 dpf morphants showed an increase in apoptosis. The fact that 40% of *atg5* morphants and less than 10% of *becn1* or *atg7* morphants survived more than 10 dpf and that the morpholino effects last less than 5 days, means that autophagy during the firsts days of development is essential for long term fish viability. *Atg5* and *atg7* showed significantly reduced numbers of autophagosomes at 3 dpf throughout the entire heart in comparison with wild type embryos. Histology of 3 dpf morphant hearts revealed enlarged atria with an accumulation of blood cells and incorrect looping compared with controls. The use of a reporter line that labels endothelial cells, including the vasculature, blood cells, and endocardial cells, showed that autophagy morphants had no vasculature defects at 3 dpf. Misexpression of *vcan* (Versican), *bmp4* (bone morphogenetic protein 4), and *notch1b* (notch receptor 1B)—genes that are necessary for atrioventricular valve development—was reported in morphants (mainly in the ventricle), while in controls the expression of these genes was confined to the atrioventricular valve. These data suggest that autophagy gene regulatory networks are necessary for correct cardiac morphogenesis. Microarray analysis of morphant hearts confirmed the altered expression of decisive genes for heart development and function. Most of the altered genes in autophagy morphants were transcriptional regulators or had roles in fundamental cellular pathways, such as the cell cycle, metabolism, and mitosis [72].

Interestingly, *atg5* function has been also shown to be necessary for caudal fin regeneration. *Atg5* knock-down was able to block regeneration of the caudal fin and induce degeneration of the existing blastema in adult zebrafish 2 days after caudal fin amputation. *Atg5* defective fin tissues showed increased apoptosis and reduced proliferation. A preosteoblast marker and osteocalcin showed impaired expression in *atg5* morpholino treated fin tissues, which shows that cell differentiation was also compromised [73]. *Atg5* function during regeneration was also assessed in a zebrafish model of extraocular muscle regeneration, in which the lateral rectus muscle was surgically removed [74]. Delivery of an *atg5* morpholino by electroporation significantly reduced muscle regeneration [74]. These studies indicate that Atg5 plays a key role in tissue regeneration.

In zebrafish models of polycystin-2 deficiency and polycystic kidney disease, co-injection of an *atg5* morpholino led to an increase in the cystic phenotype [75,76].

Finally, in a zebrafish model of Parkinson’s disease, depleted *atg5* expression was shown to aggravate the Parkinson’s disease phenotype, with a further reduction in the number of dopaminergic neurons and a worsening of locomotor behavior. In contrast, *atg5* overexpression in this model restored the number of dopaminergic neurons at levels that were equal to those of the wild type and led to recovery of locomotion. Knock-down of *atg5* caused an upregulation of β-syn, (β synuclein), PINK1 (PTEN induced kinase 1) and parkin proteins, which have been related to Parkinson’s disease. These data suggest that *atg5* is probably involved in the transcription of genes related to Parkinson’s disease and neurodegeneration [77].

*Atg5* has a role during development, autophagy, heart function, regeneration and neural development in zebrafish. As with other morphant models, it is important to confirm its role in mutant models, which could also be used to search for treatments.

- Spastic paraplegia type 76 (SPG76).

Mutations in *CAPN1* (calpain-1) have been associated with SPG76, spastic paraplegia accompanied with ataxia and spastic ataxia. *CAPN1* encodes calpain-1 catalytic subunit, which has an essential role in neuronal plasticity, migration and microtubular regulation [34].

*CAPN1* is present in zebrafish in two forms: *capn1a* and *capn1b*. Morphants for both calpain genes in zebrafish have been developed. *Capn1a*—but not *capn1b*—morphants resulted in several developmental defects at 2 dpf, such as: a hydrocephalic brain, small eyes, yolk sac extension, pericardial edema, and an aplastic swim bladder. mRNA rescue with human *CAPN1* failed because human protein was detected at 24 hpf, but not at 48 hpf, while only the zebrafish protein was detected later at 48 hpf. Examination of the CNS revealed motor neuron disorganization, migration defects of the trigeminal motor nucleus and of the facial branchiomotor neuronal cell bodies, which had not fully migrated from rhombomere 4 to 6. Moreover, the vagal motor neurons had an abnormal positioning and spacing, suggesting defects in cell motility. Disruption in the microtubule network in optic tectum and cerebellum was also observed. Furthermore, microtubules in the motor neuron axons were thinner and more disorganized [78].

This morpholino study seems to demonstrate that *capn1* has a critical role in neural function, but it is still necessary to analyze the effects of *capn1* mutations on locomotion. Moreover, the development of a mutant line is always crucial to confirm a morpholino phenotype.

- Spinocerebellar ataxia autosomal recessive type 17 (SCAR17).

A splice mutation in the *CWF19L1* (CWF19 like cell cycle control factor 1) gene was found in a Turkish family [35].

*Cwf19l1* zebrafish morphants displayed reduced locomotion and an abnormal touch-evoked escape response at 3 dpf. Defects in cerebellar structure were also observed [35].

*Cwf19l1* morphants reproduced manifestations similar to those observed in patients, but as was remarked before, it would be interesting to develop a mutant model to verify these morpholino discoveries and search for potential treatments for this rare disease.

- EAST (epilepsy, ataxia, sensorineural deafness and tubulopathy) syndrome.

Mutations in *KCNJ10* (potassium channel, inwardly rectifying subfamily J member 10), which encodes a potassium channel, cause EAST (epilepsy, ataxia, sensorineural deafness and tubulopathy) syndrome (also called SeSAME syndrome) [36,37].

Morphants for the two *KCNJ10* genes in zebrafish, *kcnj10a* and *kcnj10b*, have been generated. *Kcnj10a* morphants were shown to have a normal phenotype but demonstrated a statistically significant increase in the frequency of spontaneous contractions at 30 hpf. Abnormal frequency of spontaneous contractions was rescued upon co-injection of human *KCNJ10* mRNA, but not with human mRNA containing a mutation associated with EAST syndrome. *Kcnj10a* morphants displayed a touch-evoked escape response characterized by frequent circling or loops around their vertical axis. Locomotion was aberrant with *kcnj10a* morphants attempting to maintain an upright posture, with excessive fin movements that did not accompany locomotion. Moreover, *kcnj10a* morphants had an increased frequency of abnormal movements of the eyes and jaw. Aberrant locomotion and abnormal facial movements could indicate ataxia. Sometimes, *kcnj10a* morphant embryos displayed seizures that were consistent in bursts of speed with continuous swimming, even when they hit the wall, followed by an immediate complete loss of posture. Immunohistochemistry in *kcnj10a* morphants did not reveal any defects in the central or peripheral nervous systems, meaning that locomotion defects were due to physiological defects caused by the loss of *kcnj10a*. Co-injection of *p53* morpholino did not rescue the locomotion defect. Pronephric duct was found to be dilated in *kcnj10a* morphants. Pericardial edemas were also seen and were probably induced by water retention. Injection of a fluorescent dextran was performed in order to analyze the ability of morphants to excrete water. At 24 h 91% of the water was excreted by wild type embryos, whereas the *kcnj10a* morphants were only able to excrete 26%. *Kcnj10b* morphants were smaller and displayed morphological abnormalities, however, locomotion was impossible to analyze due to their severe phenotype. Co-injection with *p53* morpholino did not rescue this severe phenotype in *kcnj10b* morphants [37].

Epilepsy was demonstrated in the same *kcnj10a* morphant at 120 hpf using an electroencephalogram. Interestingly, pentobarbitone treatment effectively suppressed the dominating seizure activity in *kcnj10a* morphants and diazepam treatment had no effect [79].

In a more recent study, co-injection of the *kcnj10a* morpholino with mRNA from patients with *KCNJ10* mutations related to autism spectrum disorder and epilepsy did not rescue the phenotype, while normal human *KCNJ10* mRNA did. Moreover, overexpression of one mutation for the autism–epilepsy phenotype produced a similar abnormal locomotor behavior [80].

Zebrafish morphant models of EAST syndrome seem to recapitulate locomotor features of the disease, revealing epilepsy and ataxia, however, sensorineural deafness and tubulopathy were not demonstrated. However, the generation of permanent mutant models will allow for studying the course of pathology at juvenile ages and not only during the embryonic stage. Interestingly, the *kcnj10a* morphants have been demonstrated to be useful for testing candidate drugs.

- Poretti–Boltshauser syndrome.

A mutation in *LAMA1* (laminin subunit alpha 1) causes cerebellar dysplasia with retinal dystrophy [38].

*Lama1* morpholinos caused a dose-dependent phenotype that started at 36 hpf and was characterized by a shortened body, an abnormal body axis curvature, and malformed eyes that often lacked lenses and had misshapen pupils. Histological analysis of *lama1* morphants revealed reduced eye size, lens degradation with lenticular bladder cells forming a deposit in the subretinal space and corneal thickening at 72 dpf but not in previous stages [81].

A *lama1* knock-out model created using ENU mutagenesis showed defects in retinal ganglion cell axon guidance, including aberrant ipsilateral and anterior projections [82]. Furthermore, the anterior and postoptic commissures were not properly formed in *lama1* mutants. Interestingly, expression analysis of axon guidance genes showed that said genes were not altered in the mutant. Increased apoptosis was revealed in the forebrain of *lama1* mutants. There were also defects in the guidance of neurons of the bilateral nuclei and neurons of the nucleus of medial longitudinal fascicle. Reticulospinal axons showed aberrant trajectories and Mauthner axons displayed pathfinding defects. Spinal motor neurons also showed multiple axon guidance errors and increased branching. Branchiomotor neurons showed failed migration but not defects in axon outgrowth. The rest of the peripheral sensory pathways were normal and neural crest cell migration was not affected [83].

Other *Lama1* zebrafish mutant models revealed impaired notochord differentiation [84] and an ocular phenotype with irregularly shaped pupils accompanied with a shortened body axis and lethality (all the homozygous larvae die before 12 dpf) [85]. Histological analysis revealed defects in lens development at 30 hpf with disrupted epithelial cell morphogenesis and an association of the membrane blebbing with the lens capsule. At 36 hpf, apoptotic cells were distinguishable in the lens, fiber cell morphogenesis and retinal lamination were delayed, and the cornea was dysplastic. *Lama1* mutants were compared with wild type embryos, in which the lens was ablated at 24 hpf. At 10 dpf *lama1* mutants and lens ablated embryos showed significantly narrowed pupils. However, anterior segment dysgenesis was more pronounced in *lama1* mutants and the cornea showed focal dysplasia in the mutants but not in lens ablated embryos. This indicates that these differences were probably caused by the loss of *lama1* and not loss of the lens. Focal adhesions were reduced in the lens epithelium and cornea of *lama1* mutants. Moreover, lama1 morpholino embryos had multiple axonal projection defects in ganglion cells within the eye. In *lama1* mutants, mispositioned photoreceptor cells in the inner retina were detected. These defects were not observed in lens ablated embryos. Furthermore, *Lama1* morphants displayed delayed endothelial cell differentiation and severe dysmorphogenesis of the hyaloid vasculature. Tissue-specific genetic mosaics were created through blastula transplantation of *lama1* mutant cells or wild type cells in the region of the embryo that gives rise to ocular tissue. Chimeras, in which a half of the cells of the eye were from donor embryos, were raised to adulthood. *Lama1* mutant chimeras showed reduced embryo survival compared to wild type chimeras. Moreover, *lama1* mutant adult chimeras displayed ocular defects, such as severe dysplasia, microphthalmia, cataracts, lens dysmorphogenesis, small irregular pupils, disrupted scleral iridophore patterning, and iridocorneal dysgenesis. Adult wild type fish with their lenses removed showed reduced eye size at 24 hpf, small irregular pupils and a dysmorphic ciliary canal but the other structures developed normally [85]. In addition, the optokinetic response of *lama1* mutants was severely reduced at 5 dpf [86].

Using a *lama2* mutant with injection of *lama1* morpholinos revealed a complete loss of laminin immunoreactivity within the myotome, a high number of detached muscle fibers and reduced muscular birefringence, which suggests a requirement for *lama* genes in early muscle attachment [87].

Furthermore, *lama1* zebrafish mutants were used to study optic cup development. Although optic cup size was normal in *lama1* mutants, time lapse analysis of the optic cup revealed disrupted morphogenesis at 12.5 hpf. The mutant optic vesicle was rounder and more symmetric along the anterior–posterior axis than in controls. The retina was disorganized, and the neural retina and retinal pigmented epithelium were not able to enwrap the lens. The lens was ovoid rather than spherical and the choroid fissure failed to form properly. Moreover, in *lama1* mutants the connection between the brain and optic cup failed to constrict. Focal adhesion recruitment in *lama1* mutants was reduced at the optic stalk furrow, however, it was increased at the lens retina interface during invagination, meaning that *lama1* has a role in the negative regulation of focal adhesion assembly during invagination. Apoptosis was observed in medial regions of the optic cup in *lama1* mutants. However, injection of *p53* morpholino failed to rescue cell death. *Lama1* was not required for protrusive cell behaviors, as *lama1* mutants displayed normal cell behaviors although retinal structure was abnormal. Cell polarity during optic vesicle evagination was observed to be disrupted in *lama1* mutants [88].

*Lama1* zebrafish morphants and mutants have been widely used to study defects in retinal, notochord and optic cup development. However, only defects in the forebrain were reported [83] and the rest of the CNS, including the cerebellum, was not broadly investigated [83]. Future research of the CNS in these lines could reveal if they represent an appropriate model to study cerebellar dysplasia or if they are only useful for studying retinal dystrophy.

- Mitochondrial recessive ataxia syndrome (MIRAS).

*POLG* (mitochondrial DNA polymerase gamma) mutations cause MIRAS [39,40].

A zebrafish *polg* mutant model showed significantly reduced mitochondrial DNA (mtDNA) levels, starting at 1 week post fertilization (wpf) and remaining severely reduced until their death prior to 4 wpf. Reduced levels of Vdac1 (voltage dependent anion channel 1)—a protein found on the outer mitochondrial membrane—were revealed in *polg* mutant larvae, which could be related to a lower mitochondrial mass. Despite reduced mitochondrial mass, ATP levels and static lactate levels, which indicate glycolysis, were not affected in *polg* mutants. *Polg* mutant larvae were smaller, thinner and had smaller eyes than wild type larvae and *polg* heterozygous mutant larvae. Moreover, *polg* mutants also displayed deficient fin regeneration after amputation at 2 wpf. An in vivo mtDNA polymerase activity assay revealed that *polg* mutants were unable to activate mtDNA replication since mtDNA remained depleted. Interestingly, the relative abundance of mtDNA was found to be higher in the tail tissue than in the gills, heart, internal organs and CNS tissues in all genotypes. Moreover, the CNS tissue levels of mtDNA were lower compared to those of the gills, heart and internal organs. *Polg* mutant mtDNA levels were significantly reduced in all of the analyzed tissues, with the organ tissues being the most significantly affected and the tail tissues being less affected, as compared with the wild type and heterozygous mutants. The oxygen consumption rate measurements showed that the mitochondria in *polg* mutants maintained similar levels of spare respiratory capacity. However, the respiratory basal levels of homozygous *polg* larvae of 3 wpf were reduced in the CNS, with a low respiratory output [89].

Recently, Facchinello and colleagues [90] developed *polg* morphants and knock-out mutants with ENU and CRISPR/Cas9. However, the *polg* CRISPR/Cas9 line did not survive until adulthood and was less studied. Reduced body length was observed in these three zebrafish models. *Polg* morphants had an enlarged cardiac atrium and demonstrated an alteration of pathways involved in mitochondria–nucleus retrograde signaling. *Polg* knock-out ENU displayed cardiac defects (dilated heart and reductions in the trabecular network and its thickness), gonadal defects in both sexes (alteration in follicle maturation and smaller testes with reduced sperm levels) and had a reduced liver size. Birefringence assays in *polg* ENU mutants revealed defects in skeletal muscle. Moreover, an altered mitochondrial distribution, a reduction in mitochondrial mass and mtDNA expression, and a decreased oxygen consumption rate and mitochondrial superoxide production were also observed in the same model. Alterations of mitochondria–nucleus retrograde signaling were also observed in both mutant lines. In addition, *polg* CRISPR/Cas9 mutants developed early defects in skeletal muscle and showed altered responsiveness to light stimuli at 15 dpf. Finally, clofilium tosylate (a potassium channel blocker) treatment of *polg* ENU mutant larvae restored mtDNA levels. Treatment of adult *polg* mutants efficiently rescued complex I respiratory activity, mitochondrial mass and skeletal muscle defects. In *polg* morphants, cardiac frequency and atrium size was also restored by treatment with clofilium tosylate [90].

*Polg* morphants and mutant zebrafish lines show characteristics of mitochondrial disease. However, the only reference to the CNS in these studies was altered locomotion and the presence of reduced respiratory levels, which may be indicative of neuronal damage. On the other hand, treatment with clofilium tosylate seemed to alleviate part of the organ damage due to *polg* depletion. Future analysis of this model will reveal if defects exist in the CNS that recapitulate the neural damage observed in MIRAS.

- Pancreatic and cerebellar agenesis (PACA).

Mutations in *PTF1A* (pancreas transcription factor 1alpha) cause PACA [41].

The *Ptf1a* gene in zebrafish has been broadly studied, especially in pancreas development. Downregulation of *ptf1a* by morpholinos showed no gross morphological phenotype but led to a lack of differentiated exocrine cells and reduced trypsin expression. However, the morpholino did not affect the expression of the endocrine markers insulin, glucagon and somatostatin [91]. At 80 hpf, *ptf1a* morphants lacked an exocrine pancreas and they only had a rudimentary pancreatic duct. At this stage, carboxypeptidase A-producing cells had no detectable expression, although islet organization was normal. Moreover, *ptf1a* morphants had fewer anterior endocrine cells, which are located in the exocrine pancreatic parenchyma [92]. A hypomorphic *ptf1a* zebrafish mutant model showed delayed ventral pancreas specification, loss of exocrine cells and an increase in ectopic endocrine cells. These data indicate that *ptf1a* is involved in endocrine and exocrine fate specification [93].

Interestingly, *ptf1a* was also seen to play an important role in retinal development. Knock-down of *ptf1a* caused a reduction in the number of horizontal and amacrine cells. This reduction in cell numbers was not caused by increased apoptosis. However, cells that normally become amacrine and horizontal cells, changed their inhibitory neuronal fate into excitatory cell types, such as photoreceptors, bipolar, and ganglion cells, the numbers of which were significantly increased [94]. Injection of *ptf1a* morpholino into a *ptf1a* mutant line with fewer amacrine cells led to a total lack of amacrine cells. Despite the lack of amacrine cells, phalloidin staining indicated that an F-actin-rich inner plexiform layer-like neuropil was still able to form in these retinas, although it was thinner than in controls [95]. These studies using zebrafish models reveal an important role for *ptf1a* in the specification of retinal cells.

Defects in the brain of zebrafish were not reported in *ptf1a* morphants and mutants (see previous paragraphs). However, the generation of a transgenic *ptf1a* zebrafish line suggested that transcription factors and enhancers not only regulate the activation of Ptf1a expression in the developing pancreas, but also in the hindbrain, spinal cord and cerebellum [96]. New studies should focus on the use of these or similar zebrafish models to decipher the role of Ptf1a in the brain and spinal cord.

- Gordon Holmes syndrome.

Mutations in the *RNF216* (ring finger protein 216) gene alone or in combination with mutations in *OTUD4* (OTU deubiquitinase 4), which are involved in ubiquination, are the cause of Gordon Holmes syndrome [42].

Knock-down of the *rnf216* gene in zebrafish via the use of morpholinos resulted in a reduction in the size of the eye cup and optic tecta, disorganization of the cerebellum and a slight reduction in head size. This phenotype was rescued by co-injection with human *RNF216* mRNA but not with the human mRNA with mutations related to Gordon Holmes syndrome. Knock-down of zebrafish *otud4* also resulted in a reduction in the size of the optic tecta and cerebellum. Co-injection of *rnf216* and *otud4* morpholinos produced a more severe cerebellar phenotype, accompanied with an increased reduction in the size of the optic tecta and marked microphthalmia. Co-injection of human *RNF216* or *OTUD4* mRNA in double morphants rescued the phenotype, but this was not the case for co-injection of mutant *RNF216* or *OTUD4* mRNA alone. These data suggest that epistatic interactions between these mutations contribute to the disease phenotype [42].

Zebrafish morphants of *rnf216* and *otud4* showed cerebellar damage characteristic of Gordon Holmes syndrome that should be reproduced in a zebrafish mutant model. The presence of cerebellar abnormalities indicates that these might be interesting models to search for a possible treatment for this syndrome, such as the possibility of performing an unbiased drug screen.

- Childhood-onset neurodegeneration with ataxia, dystonia, and gaze palsy (NADGP).

Biallelic mutations in the *SQSTM1* (sequestosome 1) gene, which is required for intracellular signaling, the oxidative stress response, apoptosis and autophagy, cause NADGP. Dominant mutations in *SQSTM1* have been also related with Paget’s disease of the bone, amyotrophic lateral sclerosis and frontotemporal dementia (ALS/FTD) [43].

Knock-down of the *sqstm1* gene in zebrafish led to abnormal motor behavior consistent with a reduced touch-evoked escape response. Motor neuron axons were seen to suffer disrupted arborization and shortening. However, there was no neuronal loss or Mauthner cell defects in *sqstm1* morphants. Co-injection of human *SQSTM1* mRNA rescued the phenotype but this was not rescued by the co-injection of human *SQSTM1* mRNA with ALS/FTD mutations. Moreover, overexpression of wild type and mutant human mRNAs did not reproduce the morphant phenotype. As *SQSTM1* is known to regulate the activity of mTOR (regulates responses to DNA damage), mTOR levels were measured in *sqstm1* morphants and increased levels of mTOR were found. Treatment with rapamycin (an mTOR inhibitor) at 48 hpf resulted in amelioration of the motor phenotype of *sqstm1* morphants [97].

In addition, *sqstm1* knock-down in a splice site related with cerebellar ataxia, induced structural cerebellar defects in 60% of the embryos which that were specific (as no other phenotypic defects were found). These cerebellar defects ranged from depletion of the axonal connections across the midline of the cerebellum to complete atrophy. These cerebellar phenotypes were rescued by the co-injection of human *SQSTM1* mRNA, but not with the co-injection of human *SQSTM1* mRNA with disease mutations [43].

The presence of locomotor defects and cerebellar abnormalities make these morpholinos interesting for future studies that compare these findings with new mutant models and to search for treatments that can rescue these specific phenotypes.

- Spinocerebellar ataxia autosomal recessive type 7 (SCAR7) and ceroid lipofuscinosis neuronal 2 (CLN2).

TPP1 (tripeptidyl peptidase 1) is a serine protease mainly expressed in the lysosome and melanosome. *TPP1* mutations have been linked with SCAR7 and CLN2. It has been proposed that loss of function variants with abolished TPP1 enzyme activity leads to CLN2 disease, whereas variants that diminish TPP1 enzyme activity lead to SCAR7 [44].

Morpholino knock-down of *tpp1* in zebrafish causes severe developmental defects starting at 28 hpf with an abnormal head and increased apoptosis throughout the body. At 52 hpf, *tpp1* morphants developed curly tails and cardiovascular defects such as cardiac loop malformation, pericardial edema, reduced cardiac contractility and low blood flow through the heart [98].

More recently, a knock-out of the *tpp1* gene was observed to recapitulate the pathological and behavioral features of CLN2 disease. *Tpp1* mutant zebrafish defects started at 48 hpf with a curved body, reduced head and a smaller retina. At 72 hpf, there was no detectable jaw and at later stages the swim bladder was not present in *tpp1* mutants. In addition, *tpp1* mutants died prematurely before 7 dpf. Stored levels of subunit c of mitochondrial ATP synthase in lysosomes, which is a characteristic of CLN2 disease, were observed to be increased in the whole body including the eye, head, spinal cord, and most prominently, the muscle fibers in 48 hpf *tpp1* mutants. CNS examination revealed a deep absence of HuC/D-positive differentiated neurons in the retina, optic tectum and cerebellum of *tpp1* mutants and *tpp1* morphants. Moreover, astrocytosis was observed in the thalamus and cerebellum of *tpp1* mutants at 48 hpf. Selective apoptosis was seen in the retina, optic tectum, cerebellum and spinal cord in *tpp1* mutants at 48 hpf. This selective apoptosis is similar to that found in human patients of CLN2 disease. Decreased proliferation was shown at 48 hpf in the retina, forebrain, midbrain–hindbrain boundary and spinal cord of *tpp1* mutants and *tpp1* morphants. Axonal disorganization, which is another feature of CLN2 disease, was shown to start at 48 hpf in *tpp1* mutants and *tpp1* morphants. Axonal disorganization in *tpp1* deficient zebrafish was characterized by posterior commissure defasciculation, absence of the trochlear nerve and almost complete absence of the optic nerve, which failed to send fibers to the optic tectum. No axonal defects were seen before 48 hpf and primary axon tract formation was not affected in *tpp1* deficient models, meaning that axonal loss was secondary to degenerative changes. Locomotion was impaired in *tpp1* mutants at 72 hpf. Mutants had an aberrant swimming pattern demonstrating many turns, a significantly increased hyperactive behavior covering long distances, and seemed to show convulsions. At 96 hpf, *tpp1* mutants displayed sustained muscle contractions and lost the ability to move. The touch-evoked escape response was also reduced in *tpp1* mutants [99]. Seizure activity in *tpp1* mutants was demonstrated through single electrode electroencephalography. Treatment with valproate, but not with pentobarbitone, significantly reduced seizure-related movement and reduced the mortality between 3 and 6 dpf [100]. This exhaustive research and the positive results obtained with valproate open up the possibility of performing high throughput drug screen against CLN2 in *tpp1* zebrafish mutants.

- Spinocerebellar ataxia autosomal recessive type 24 (SCAR24).

Biallelic mutations in the *UBA5* (ubiquitin-activating enzyme of UFM1) gene were recently seen to cause SCAR24 [45] and early onset encephalopathy with intellectual deficiency, microcephaly, movement disorders, and epilepsy [101]. Studies in fibroblasts from affected individuals showed that *UBA5* mutations impaired the process of UFMylation, resulting in an abnormal endoplasmic reticulum structure [101].

Injection of *uba5* morpholinos in zebrafish embryos did not result in a morphological phenotype. However, locomotion defects were observed in these morphants. *Uba5* morphants had reduced movements and difficulties to exit the chorion in comparison with wild type larvae. The touch-escape response of *uba5* morphant larvae at 72 hpf was decreased. Larvae exhibited looping or pinwheel swimming which resembled a seizure-like behavior. The spontaneous motility of *uba5* larvae at 5 dpf was observed to be severely affected [101].

The next necessary step will be to develop a *uba5* zebrafish mutant line to reveal whether it recapitulates the *uba5* morpholino locomotion defects. More research is also needed to understand the cellular/molecular/physiological defects caused by *uba5* knock-down.

- Galloway-Mowat syndrome.

Galloway-Mowat syndrome is caused by mutations in the *WDR73* (WD repeat domain 73) gene, which has high levels of expression in the brain, mainly in the cerebellum [46].

A Galloway-Mowat syndrome zebrafish model was generated using *wdr73* morpholinos. *Wdr73* morphants demonstrated a reduced head size, brain morphology defects, hypopigmentation—which displayed incomplete penetrance—and a curved body or truncated tail region at 1–2 dpf. *Wdr73* morphants lacked expression of *dmbx1a*, which is related to midbrain progenitor cells, in the cerebellum at 48 hpf but not at 24 hpf. At 48 hpf, *wdr73* morphants had a poorly expanded and differentiated midbrain. However, *fgf8* (fibroblast growth factor 8) expression, which is a marker of progenitor cells of the midbrain-hindbrain boundary, was equal to that of wild type embryos. Morphogenesis of the midbrain and hindbrain was disrupted in *wdr73* morphants, leading to dilated ventricles. Proliferation of progenitor cells was also reduced in *wdr73* morphants at 1 dpf. Purkinje cells in *wdr73* morphants were absent at 4 dpf. This cerebellar phenotype was rescued in all the larvae co-injected with zebrafish *wdr73* mRNA and in the majority of larvae co-injected with human *WDR73* mRNA. In contrast, co-injection with human mRNA containing a nonsense mutation related to Galloway-Mowat syndrome did not rescue the phenotype [46]. As was previously mentioned for other morpholino models, the creation of mutant lines that recapitulate the cerebellar phenotype is fundamental to progress research in this area, especially when morpholinos cause severe morphological defects such as in this case.

- Spinocerebellar ataxia autosomal recessive type 12 (SCAR12).

SCAR12 is produced by mutations in the *WWOX* (WW domain containing oxidoreductase) gene, which has functions in DNA repair and acts as tumor suppressor gene [48].

Tsuruwaka and colleagues used morpholinos and siRNA to knock-down zebrafish *wwox* to study intracellular Ca^2+^ dynamics. *Wwox* knock-down caused severe edemas, curled backbones, reduced body length, head and eye size and increased lethality in embryos. In addition, these models had altered Ca^2+^ dynamics [102]. This model presented developmental delay, which is a characteristic of SCAR12 and also had disturbed Ca^2+^ dynamics, which remarks the *wwox* role in DNA repair. However, the CNS was not studied and locomotion assays to evaluate epilepsy were not performed. Future studies looking at these aspects will inform us as to whether zebrafish can be a good model for SCAR12.

### 3.3. Recessive Inherited Disorders Related to Ataxia

In this section we discuss several zebrafish models designed to model pathologies that present ataxia as one of their symptoms: Niemann–Pick disease type C (NPC), CAMRQ3, Joubert syndrome and pontocerebellar hypoplasia.

- Niemann–Pick disease type C (NPC).

NPC is a rare neurodegenerative lysosomal storage disorder caused by mutations in the *NPC1* (95% of the cases) or *NPC2* genes, which are involved in cholesterol trafficking. The clinical diagnosis requires fibroblast staining with filipin to determine whether accumulation of unesterified cholesterol in lysosomes is present [50].

Morpholino knock-down of *npc1* in zebrafish led to developmental delay characterized by a reduced progression of epiboly. Co-injection with *Npc1* mouse mRNA rescued the epiboly delay. When the *npc1* morpholino was injected in the yolk syncytial layer at 1000 cell stage, epiboly was delayed more often. This means that *npc1*, expressed in the yolk syncytial layer, is critical for normal epiboly movements. Analysis of mesoderm induction and cell fates revealed that both develop normally in *npc1* morphants. Actin cytoskeleton disorganization was also observed in *npc1* morphants, with actin clumping in some cells and localized loss of actin microfilaments in other cells. Treatment with the steroid hormones pregnenolone and Dex in *npc1* morphants partially rescued epiboly delay and actin cytoskeleton defects, suggesting that a reduction in steroidogenesis was, in part, responsible for the epiboly delay phenotype in *npc1* morphants. *Npc1* morphants that completed epiboly presented a phenotype characterized by shorter body axis, wider notochord and somites and died before 2 dpf. Apoptosis increased in *npc1* morphants at 1 dpf. In addition, *npc1* morphants displayed punctate sterol distribution in cells after filipin staining at 12 hpf [103].

Since thrombocytopenia is found in some patients with NPC1, this symptom was also studied by Louwette et al. [104] in *npc1* morphants. Zebrafish injected with *npc1* morpholinos had malformed heads and dysmorphic brains and eyes. Filipin staining revealed defective intracellular processing of cholesterol. Study of thrombocytes revealed that, even with a low dose of morpholino, 82% of the embryos had an almost complete absence of thrombocytes in the caudal hematopoietic tissue at 3 dpf. When the dose was doubled, 90% of the embryos had thrombocytopenia and died at 5 dpf. Erythrocytes numbers were significantly reduced in *npc1* morphants [104].

The generation of a *npc1* stable mutant model using CRISPR/Cas9 was able to reproduce some of the symptoms of human patients. *Npc1* mutant zebrafish had a reduced lifespan; the majority of them died before 2 months post-fertilization and none of them survived after 8 months. Moreover, *npc1* mutants had a reduced body length and exhibited ataxia symptoms. Histological analyses allowed for the observation of macroscopic hepatomegaly and splenomegaly with foamy and vacuolated liver cells. Lipid accumulation in the livers of *npc1* mutants and a massive accumulation of cholesterol in hepatocytes was also observed. A lipid profile analysis of liver tissues revealed significant differences in *npc1* mutants in profiles of ceramide, diacylglycerol, lysophosphatidic acid, phosphatidic acid, phosphatidylcholine, phosphatidyl ethanolamine, phosphatidyl serine and triglyceride, as compared to wild types. Interestingly, seven ceramides—which are characteristic of NPC1 disease—were accumulated in the liver of *npc1* mutants. Finally, calretinin expression was significantly reduced in the cerebellum of *npc1* mutants at two months post-fertilization, which indicates a loss of Purkinje cells [105].

Tseng et al. [106] also generated a CRISPR/Cas9 *npc1* model that had reduced lifespan (died before 6 months of age), infertility and reduced body length compared to wild type fish. Moreover, adult *npc1* mutants failed to maintain balance during swimming and died a few days after the balance defect was observed. Histopathological analyses revealed axonal spheroids in the hindbrain of adult *npc1* mutants and disorganized Purkinje neurons in the cerebellum. The first sign of liver disease appeared at 7 dpf in *npc1* mutants. They had bigger, dark and opaque livers as compared to wild type larvae. Histopathological analysis of liver sections showed larger hepatocytes full of vacuole like structures in *npc1* mutants. In addition, filipin staining demonstrated cholesterol accumulation in *npc1* mutant liver tissue. Surprisingly, lipid accumulation was not observed in *npc1* mutants. Filipin positive staining started at 2 dpf in *npc1* mutants in the yolk area and at 3 dpf in lateral line tissues. By 5 and 7 dpf, *npc1* mutants accumulated more unesterified cholesterol throughout the entire trunk area. Cholesterol accumulation was observed along the notochord and the ventral edge of the trunk in *npc1* mutants. Application of a pCS2+ plasmid with *npc1* cDNA and EGFP rescued the unesterified cholesterol accumulation in the yolk area. Lysosomes in live larval zebrafish were observed using LysoTracker Red staining. At 5 dpf, *Npc1* mutants had a stronger LysoTracker Red staining signal compared to wild type larvae, mainly in the neuromasts and in the olfactory placode of *npc1* mutants at 3 dpf. Based on these results, LysoTracker Red staining was used to test drug efficacy in vivo. The 2HPβCD compound, which was seen to be effective in other animal models and had been tested in humans, was tested on 3 dpf larvae. After 3 days of treatment, a significant reduction in LysoTracker Red staining was observed in *npc1* mutants. Filipin staining also revealed reduced accumulation of cholesterol in *npc1* mutants. However, the 2HPβCD treatment did not rescue the liver defects or improve survival [106]. In any case, the development of a method to test compounds in *npc1* mutant zebrafish is crucial for attempting to discover new therapies for NPC.

- Cerebellar ataxia and mental retardation with or without quadrupedal locomotion 3 (CAMRQ3).

Mutations in carbonic anhydrase 8 (*CA8*) are related to CAMRQ3 [51]. CA8 is a catalytically inactive isoform belonging to the zinc-containing metalloenzymes family, that catalyzes the reversible hydration of carbon dioxide [52].

The *ca8* gene was found to be strongly expressed in the zebrafish nervous system, and specifically, in the Purkinje cells, which correlates with the observed expression in Purkinje cells in humans and mice. *Ca8* morpholinos caused defects in the zebrafish head as early as 9 hpf and increased mortality. At 1 dpf, *ca8* morphants had defects in the head, a fragile body, curved tail, small eye size and pericardial edema that were dose-dependent [52,107]. As development advanced, defects such as the shortened tail, curved body axis, absence of swim bladder became more obvious. The swimming pattern was altered even with a low morpholino dose. *Ca8* morphants swam at a slower speed, showed an increased turning angle, and most of them swam along the periphery of the Petri dish. Furthermore, *ca8* morphants had a pronounced difficulty in balancing the body while swimming compared with wild type. When the concentration of *ca8* morpholino was increased, the larvae progressively lost their ability to swim, until higher doses caused a complete loss. The co-injection of a *p53* morpholino did not rescue the phenotype defects or mortality rates. Gross morphological changes in the cerebellar region and a reduction in the size of the cerebellum was observed in *ca8* morphants. Moreover, *ca8* morphants showed abnormal muscle development and apoptosis in the head region and periphery of the tail region [52]. Touch-evoked responsiveness was also reduced in *ca8* morphants due to defective cerebellar function, as no defects were observed in the motor neurons or Rohon Beard sensory neurons [107]. *Ca8* zebrafish morphants seem to reproduce the typical features observed in CAMRQ3. As with other disorders, it would be of interest to develop a stable zebrafish mutant model that reproduces these (or similar) phenotypes.

- Joubert syndrome.

Joubert syndrome consists of a group of early onset recessive or X-linked cerebellar ataxias with a characteristic mid-hindbrain malformation called the molar tooth sign [53]. More than 30 genes have been associated with Joubert syndrome. Several zebrafish models have been created to study some of these genes, but most of them were focused solely on their function in ciliogenesis or retinal development [108,109,110,111,112,113,114].

Mutations in the *AHI1* (Abelson helper integration site 1) gene are the most common cause of Joubert syndrome. Knock-down of *ahi1* in zebrafish caused a curved body and abnormalities in the development of the eye, hindbrain (hydrocephalus) and otoliths, together with defects in cyst formation in the pronephric kidney tubules. These malformations were comparable to those of patients with Joubert syndrome. Co-injection of murine *Ahi1* mRNA efficiently rescued the phenotype. Histological analyses revealed defects in lamination of the cell layers of the retina in *ahi1* morphants. Reversed cardiac looping with the heart in a L-loop pattern was found in *ahi1* morphants. Moreover, cloacal dilatation and loss of pronephric cilia was observed in the pronephric ducts [115].

More recently, Lessieur et al. [116] generated an *ahi1* TALEN mutant model and showed that mutants had cone degeneration and rhodopsin mislocalization in rods at 5 months of age as well as absence of distal pronephric duct cilia [116]. Zhu et al. [117] used zebrafish morpholinos and CRISPR/Cas9 mutants to analyze if *ahi1* mutations lacking the intact WD40 repeats affect axonal decussation. They found that *ahi1* morphants and mutants had retinal ganglion cell axon misprojection and ocular dysplasia caused by a toxic gain of function [117].

Other zebrafish models of different genes related to Joubert syndrome, such as a knock-down model of *cep290* (centrosomal protein 290), also recapitulate features of Joubert syndrome, such as cerebellar abnormalities, hydrocephalus, retinal defects, abnormalities in otic cavity development and pronephric cysts [118,119]. Moreover, *cep290* morphants were used to search for therapies for ciliopathic renal disease, which resulted in the discovery that rapamycin and roscovitine ameliorate the symptoms [120]. More recently, a mutant line was developed to study retinal degeneration. *Cep290* mutants presented scoliosis and progressive cone deterioration [114].

Similarly, knock-down of *cspp1* (centrosome and spindle pole associated protein 1), *cep104* (centrosomal protein 104), *kiaa0556* (katanin interacting protein) and *poc1b* (proteome of the centriole 1B) in zebrafish resulted in a ciliopathy phenotype, which is characteristic of Joubert syndrome [121,122,123,124,125,126].

Most of these Joubert syndrome zebrafish models were not used to study the CNS. In addition, most of them were not created with mutants that we suspect would probably reproduce better the traits of this syndrome.

- Pontocerebellar hypoplasia.

Pontocerebellar hypoplasia constitutes a group of early onset rare neurodegenerative disorders with variable symptoms of cerebellar ataxia. Several mutations in different genes have been associated with different forms of pontocerebellar hypoplasia. Mutations in *TSEN54*, a tRNA splicing endonuclease subunit gene, were associated with pontocerebellar hypoplasia type 6 and late onset dominant hereditary ataxia [54].

Morpholino knock-down of *tsen54* in zebrafish resulted in brain hypoplasia and loss of structural integrity inside the brain with an aberrant mid-hindbrain boundary. Co-injection of human *TSEN54* mRNA partially rescued the brain phenotype. Co-injected embryos displayed milder brain hypoplasia and a more defined brain structure. Interestingly, *tsen54* morpholino abnormalities were not associated with neurodevelopmental patterning defects since expression of *fgf8* (which plays a role in the maintenance and development of the mid-hindbrain boundary) and *otx2* (orthodenticle homeobox 2) (which functions in the early regionalization of the mesencephalon) at 24 hpf was normal. *Tsen54* morphants had increased levels of apoptosis in the brain. A stable *tsen54* knock-out model created with ENU mutagenesis had a reduced survival rate with all the *tsen54* homozygous larvae dying before 9 dpf. However, the defects that caused their lethality were not studied and it is not known if mutations in *tsen54* also caused brain hypoplasia. In addition, knock-down of the *rars2* (arginyl-tRNA synthetase 2) gene, which has been also related to pontocerebellar hypoplasia type 6, resulted in a similar brain phenotype to that observed in *tsen54* morphants, which was also partially rescued by the co-injection of *RARS2* human mRNA [127]. The better characterization of the mutant model would help to study the molecular consequences of pontocerebellar hypoplasia.

Mutations in the *SLC25A46* (solute carrier family 25 member 46) gene, which plays a role in mitochondrial dynamics, have been also related to pontocerebellar hypoplasia and progressive myoclonic ataxia with optic atrophy and neuropathy [128,129].

*Slc25a46* was observed to be prominently expressed in the brain and the spinal cord during zebrafish embryonic development. Consistent with this, slc25a46 zebrafish morphants showed a neurodegenerative phenotype with a failure of the development of the midbrain and hindbrain, severe curly tail morphology and abnormal locomotion. Motor neurons had significantly shorter axon tracts and many of them failed to innervate the rostral myotome at 48 hpf, the spinal cord neuropil had fewer neuronal processes, fewer retinal ganglion cell axons reached the tectum at 72 hpf, and retinal ganglion cell dendrites were also affected. Degeneration of motor neuron terminals was also observed. *Slc25a46* zebrafish morphants presented a misallocation of the mitochondria, which were in the process of fission within the cell bodies. This suggested that incomplete fission of mitochondria in *slc25a46* morphants might inhibit transport and distribution into neuronal processes. Mitochondrial dynamics were studied in dissociated neurons from *slc25a46* zebrafish morphants. Mitochondria from morphants were longer than those of wild type embryos and were immobile. Co-injection with *SLC25A46* human mRNA restored the size of the mitochondria but not the co-injection with mutant *SLC25A46* mRNA. On the other hand, overexpression of the wild type mRNA resulted in mitochondrial fragmentation and disruption of the mitochondrial network [129,130].

In contrast to the morphant studies, a CRISPR/Cas9 *slc25a46* zebrafish model showed no phenotype because of genetic compensation, as was demonstrated by RNA sequencing. Injection of a *slc25a46* morpholino into an F2 homozygous *slc25a46* zebrafish CRISPR/Cas9 line did not cause a phenotype. However, F0 embryos injected with an *slc25a46* CRISPR/Cas9 construct had a phenotype that resembled *slc25a46* morphants since they had smaller eyes, heart edema, a shorter trunk and an increase in motor neuron axon disruptions in comparison with wild types. The F0 *slc25a46* CRISPR/Cas9 phenotype was rescued after the co-injection of *SLC25A46* human mRNA. The existence of a phenotype in *slc25a46* CRISPR/Cas9 mosaics of the F0 generation, but not in the homozygous F2 generation, indicates that the genetic compensation starts after the F0 generation. RNA sequencing showed a high number of serine proteases and their transcription factors, serine protease inhibitors, DNA-binding proteins and a smaller number of transporters, proteins related to immunity that were expressed differentially in F2 *slc25a46* CRISPR/Cas9 embryos at 48 hpf [131]. In this case, it seems that the *slc25a46* morphant model better represents the main features of pontocerebellar hypoplasia caused by mutations in *SLC25A46* (and not the mutant homozygous model that suffers genetic compensation). This morphant model will help to further study pontocerebellar hypoplasia and search for possible treatments.

## 4. Conclusions

The majority of the zebrafish models discussed here for genes related with recessive ataxias reproduced some of the neuronal and non-neuronal phenotypes observed in human disorders. The models that did not develop any neuronal symptoms or locomotor deficits were: *atm* morphants and mutants [55,56]; *polg* mutants [89,90]; *ptf1a* morphants, mutants and transgenic lines [91,92,93,94,95,96]; *wwox* morphants and siRNA models [102]. In any case, *atm* models developed other non-neuronal symptoms of AT such as radiosensitivity, infertility and immunodeficiency [55,56]. *Polg* mutants, which were analyzed in order to study mitochondrial disease, showed reduced mitochondrial numbers, reduced respiratory levels in the CNS, and altered locomotion [89,90]. *ptf1a* models reproduced the pancreatic agenesis of PACA [91,92,93,94,95,96]. *wwox* knock-down models, which demonstrated developmental delay, were only used to study Ca^2+^ dynamics, highlighting the role of *wwox* in DNA repair [102]. In addition, *snx14* mutants [61] did not recapitulate the *snx14* morphant motor neuron deficit phenotype [27], but revealed a role for this gene in lipid biogenesis [61].

Most of the publications of autosomal recessive ataxias employed morpholinos to cause a knock-down and a loss of function effect. The morpholino effect is transient, its degradation occurs in few days and can sometimes cause off-target effects. For this reason, it is fundamental to validate it with morpholino controls (standard or mismatch morpholinos), PCR/Western blot/immunohistochemistry and mRNA rescue. As stated above for several models, stable mutant lines are necessary to complement morpholino studies because they are more informative and might facilitate drug screening [62]. However, mutant models do not always recapitulate the phenotypes observed with morphants due to genetic compensation and maternal mRNA rescue.

Although zebrafish are not expected to fully reproduce the human phenotypes due to their less intricate CNS and their limited number of complex behaviors, they are very informative for unravelling gene functions and analyzing gene and molecular pathways. In addition, their small size and external fertilization simplify high throughput drug or genetic screening. In this review, we reported various drug treatments that alleviate the symptoms of zebrafish models of autosomal recessive ataxias [70,79,90,97,100,103,106,120]. Future work should apply the use of these models for high throughput drug screening.

Recent technological advances in genetic editing, such as the development of CRISPR/Cas technologies [132], will improve the number of specific stable mutant lines that better mimic human conditions, while the use of novel techniques of microscopy and imaging, such as super-resolution or light-sheet microscopy, in zebrafish will ease phenotypic characterization.

## Figures and Tables

**Table 1:** Summary of molecular mechanisms and main clinical features of the ataxias discussed in this article.

Disease	Gene	Protein Function	Clinical Features Other Than Ataxia *	References
Ataxia-telangiectasia (AT)	*ATM*	Cellular responses to DNA damage and cell cycle control	Oculocutaneous telangiectasia, radiosensitivity, predisposition to lymphoid malignancies, cellular and humoral immunodeficiency	[24]
Boucher–Neuhäuser syndrome; Spastic paraplegia type 39 (SPG39)	*PNPLA6*	Phospholipase	Hypogonadotropic hypogonadism, chorioretinal dystrophy	[25]
Marinesco-Sjögren syndrome	*SIL1*	Nuclear exchange factor for the endoplasmic reticulum resident chaperone BiP	Cataracts, skeletal muscle myopathy, developmental delay	[26]
Spinocerebellar ataxia autosomal recessive type 20 (SCAR20)	*SNX14*	Autophagosome function	Intellectual disability, coarse facial features, hearing loss, epilepsy, macrocephaly	[27]
Ataxia with isolated vitamin E deficiency (AVED)	*TTPA*	Distribution of vitamin E	Hyporeflexia, decreased vibration sense, cardiomyopath, retinitis pigmentosa	[28]
Wolfram syndrome (WFS)	*WFS1*	Membrane trafficking, endoplasmic reticulum stress and calcium homeostasis	Mental retardation, seizures, psychiatric symptoms, pigmentary retinopathy, Diabetes mellitus, optic atrophy, sensorineural hearing loss, urinary tract abnormalities	[29,30]
Polyneuropathy, hearing loss, ataxia, retinitis pigmentosa and cataract (PHARC)	*ABHD12*	Endocannabinoid and phospholipid metabolism	Cataracts, hearing loss, retinitis pigmentosa, demyelinating sensorimotor polyneuropathy	[31]
Cayman ataxia	*ATCAY*	Neural development	Hypotonia, psychomotor retardation	[32]
Spinocerebellar ataxia autosomal recessive type 25 (SCAR25)	*ATG5*	Autophagy	Mental retardation, developmental delay	[33]
Spastic paraplegia type 76 (SPG76)	*CAPN1*	Neuronal plasticity, migration, microtubular regulation	Skeletal abnormalities, peripheral neuropathy, amyotrophy, spastic paraplegia	[34]
Spinocerebellar ataxia autosomal recessive (SCAR17)	*CWF19L1*	Cell Cycle Control	Developmental delay, mental retardation	[35]
EAST syndrome	*KCNJ10*	Potassium channel	Short stature, epilepsy, sensorineural deafness, mental retardation, renal tubulopathy	[36,37]
Poretti-Boltshauser syndrome	*LAMA1*	Cell attachment, migration	Retinal dystrophy	[38]
Mitochondrial recessive ataxia syndrome (MIRAS)	*POLG*	Catalytic subunit of mitochondrial DNA polymerase	Ophthalmoplegia, polyneuropathy, myopathy, encephalopathy, liver failure, muscle pain, sensorineural hearing loss, epilepsy	[39,40]
Pancreatic and cerebellar agenesis (PACA)	*PTF1A*	Pancreatic development	Neonatal diabetes mellitus, pancreatic and cerebellar agenesis	[41]
Gordon Holmes syndrome	*RNF216*	Ubiquitination	Chorea, dementia, hypogonadotropic hypogonadism	[42]
Childhood-onset neurodegeneration with ataxia, dystonia, and gaze palsy (NADGP)	*SQSTM1*	Intracellular signalling, oxidative stress response, apoptosis and autophagy	Dystonia, gaze palsy, dyskinesia, cognitive decline	[43]
Spinocerebellar ataxia autosomal recessive type 7 (SCAR7), Ceroid lipofuscinosis neuronal 2 (CLN2)	*TPP1*	Serine peptidase	SCAR7: Slowly progressive spasticity CLN2: Rapidly progressive myoclonus, developmental regression, early death, epilepsy, loss of vision	[44]
Spinocerebellar ataxia autosomal recessive type 24 (SCAR24)	*UBA5*	Ubiquitin-like	Cataracts, demyelinating sensorimotor neuropathy	[45]
Galloway-Mowat syndrome	*WDR73*	Unknown	Intellectual disability, short stature, microcephaly, facial dysmorphism, hypotonia, nephrotic synrome, cataracts, skin hypopigmentation	[46]
Spinocerebellar ataxia autosomal recessive type 12 (SCAR12)	*WWOX*	DNA repair	Epilepsy, mental retardation, developmental delay. microcephaly	[47,48,49]
Niemann–Pick disease type C (NPC)	*NPC1*	Cholesterol trafficking	Learning difficulties, neuropsychiatric symptoms, dementia, seizures, dystonia, supranuclear gaze palsy, neonatal cholestasis, hepatosplenomegaly	[50]
Cerebellar ataxia and mental retardation with or without quadrupedal locomotion 3 (CAMRQ3)	*CA8*	Unknown	Mental retardation quadrupedal gait	[51,52]
Joubert syndrome	Several genes	Diverse functions	Hypotonia, Developmental delay, oculomotor apraxia, breathing dysregulation, retinal abnormalities, renal cysts, polydactily, hepatic fibrosis	[53]
Pontocerebellar hypoplasia	Several genes	Diverse functions	Hypoplasia of the cerebellum or the ventral pons, progressive microcephaly, motorneuron disease, resiratory insuficiency, mental retardation	[54]

* Cerebellar ataxia is a feature of all the disorders in this table. Not all listed clinical manifestations are necessarily present in every patient with the disorder. EAST syndrome: epilepsy, ataxia, sensorineural deafness and tubulopathy.

## Supplementary Materials

The following are available online at https://www.mdpi.com/article/10.3390/cells10040836/s1, Supplementary Table S1: Description of zebrafish models of autosomal recessive ataxias.
